# Monascuspiloin Enhances the Radiation Sensitivity of Human Prostate Cancer Cells by Stimulating Endoplasmic Reticulum Stress and Inducing Autophagy

**DOI:** 10.1371/journal.pone.0040462

**Published:** 2012-07-03

**Authors:** Hui-Wen Chiu, Wen-Hung Fang, Yen-Lin Chen, Ming-Der Wu, Gwo-Fang Yuan, Sheng-Yow Ho, Ying-Jan Wang

**Affiliations:** 1 Department of Environmental and Occupational Health, National Cheng Kung University, Medical College, Tainan, Taiwan; 2 Bioresource Collection and Research Center (BCRC), Food Industry Research and Development Institute, Hsinchu, Taiwan; 3 Department of Radiation Oncology, Sinlau Christian Hospital, Tainan, Taiwan; NIH, United States of America

## Abstract

Prostate cancer is a very common cancer among males. Traditional treatments for prostate cancer have limited efficacy; therefore, new therapeutic strategies and/or new adjuvant drugs must be explored. Red yeast rice (RYR) is a traditional food spice made in Asia by fermenting white rice with *Monascus purpureus* Went yeast. Accumulating evidence indicates that RYR has antitumor activity. In this study, PC-3 cells (human prostate cancer cells) were used to investigate the anti-cancer effects of ionizing radiation (IR) combined with monascuspiloin (MP, a yellow pigment isolated from *Monascus pilosus M93*-fermented rice) and to determine the underlying mechanisms of these effects *in vitro* and *in vivo*. We found that IR combined with MP showed increased therapeutic efficacy when compared with either treatment alone in PC-3 cells. In addition, the combined treatment enhanced DNA damage and endoplasmic reticulum (ER) stress. The combined treatment induced primarily autophagy in PC-3 cells, and the cell death that was induced by the combined treatment was chiefly the result of inhibition of the Akt/mTOR signaling pathways. In an *in vivo* study, the combination treatment showed greater anti-tumor growth effects. These novel findings suggest that the combined treatment could be a potential therapeutic strategy for prostate cancer.

## Introduction

Red yeast rice (RYR), also known as red Koji or “Hongqu”, comprises primarily nonglutinous rice, red yeast, and byproducts of the fermentation. RYR is a traditional food spice that is consumed throughout Asia [1]. It is produced by fermenting the food fungus, a *Monascus* species, with steamed rice and has been used for more than 600 years to produce wines and other fermented food products. Several species of the fungus *Monascus* have been widely used in making red wine and red soybean cheese [2]. *Monascus*-fermented products have many functional secondary metabolites, including monacolin K, citrinin, ankaflavin, and monascin. In several recent studies, these secondary metabolites have shown anti-inflammatory, anti-oxidative, and antitumor activities [3], [4]. Many studies have also investigated the anti-cancer capability of RYR, such as in colon, breast, lung, and prostate cancer [3]. The classes of polyketide structures that result from the fermentation process are called monacolins, and the major monacolin found in RYR is monacolin K [5]. However, RYR showed a more potent inhibition of cancer cell growth when compared with monacolin K [6], [7]. However, the other polyketides in RYR may provide a botanical approach to cancer therapy that is worthy of further investigation.

Prostate cancer is the world's most common malignancy and the second leading cause of death from cancer [8]. Early-stage prostate cancer is androgen-dependent and can be treated effectively with androgen ablation therapy, radiation, and/or surgery. However, prostate cancer cells advance to an androgen-independent state where they can progress and lead to metastasis and death [9]. Because chemotherapy and radiation therapy are largely ineffective and metastatic diseases frequently develop even after surgery, there are limited treatment options available for prostate cancer [10]. Therefore, novel methods of treating prostate cancer must be developed. Accumulating evidence has shown that acquired resistance to radiation therapy can be overcome by utilizing small-molecule compounds that target key proteins involved in radiation resistance [11]. Anticancer therapeutic approaches such as ionizing radiation (IR), can activate cellular apoptotic machinery in androgen-independent prostate cancer cells [12]. However, acquired radiation resistance can develop in hormone-refractory prostate cancer that is associated with apoptosis resistance [13]. Previous studies have also demonstrated that defects in the apoptotic machinery are correlated with the resistance of cancer cells to current therapeutic interventions, including IR [14].

Autophagy is an important catabolic process responsible for degrading and recycling long-lived proteins, cellular aggregates and damaged organelles. In addition to the well-documented role of autophagy in cell survival, a function for autophagy in cell death has long been proposed [15]. In recent years, the role of autophagy as an alternative cell death mechanism has been a topic of debate. Studies are ongoing to define optimal strategies to modulate autophagy for cancer prevention and therapy and to exploit autophagy as a target for anticancer drug discovery [16]. Activation of the PI3 kinase/Akt pathway, a well-known method of inhibiting apoptosis, also inhibits autophagy [17]. Akt phosphorylates mammalian target of rapamycin (mTOR), which has been reported to inhibit the induction of autophagy [18]. Previous studies have demonstrated that the endoplasmic reticulum (ER) stress response, in combination with autophagy, represents an adaptive mechanism for supporting cell survival in response to a great variety of detrimental conditions [19]. ER stress activates a set of signaling pathways that are collectively termed the Unfolded Protein Response (UPR). Typically, UPR signaling promotes cell survival by improving the balance between the protein load and the folding capacity in the ER [20]. However, if the cells are exposed to prolonged or robust ER stress, the cells die by apoptosis [21]. Increasing evidence has indicated that ER stress is also a potent trigger of autophagy [22]. Thus, a better understanding of the signaling pathways that control ER stress -induced autophagy and cellular fate will hopefully open new possibilities for cancer treatment.

**Figure 1:**
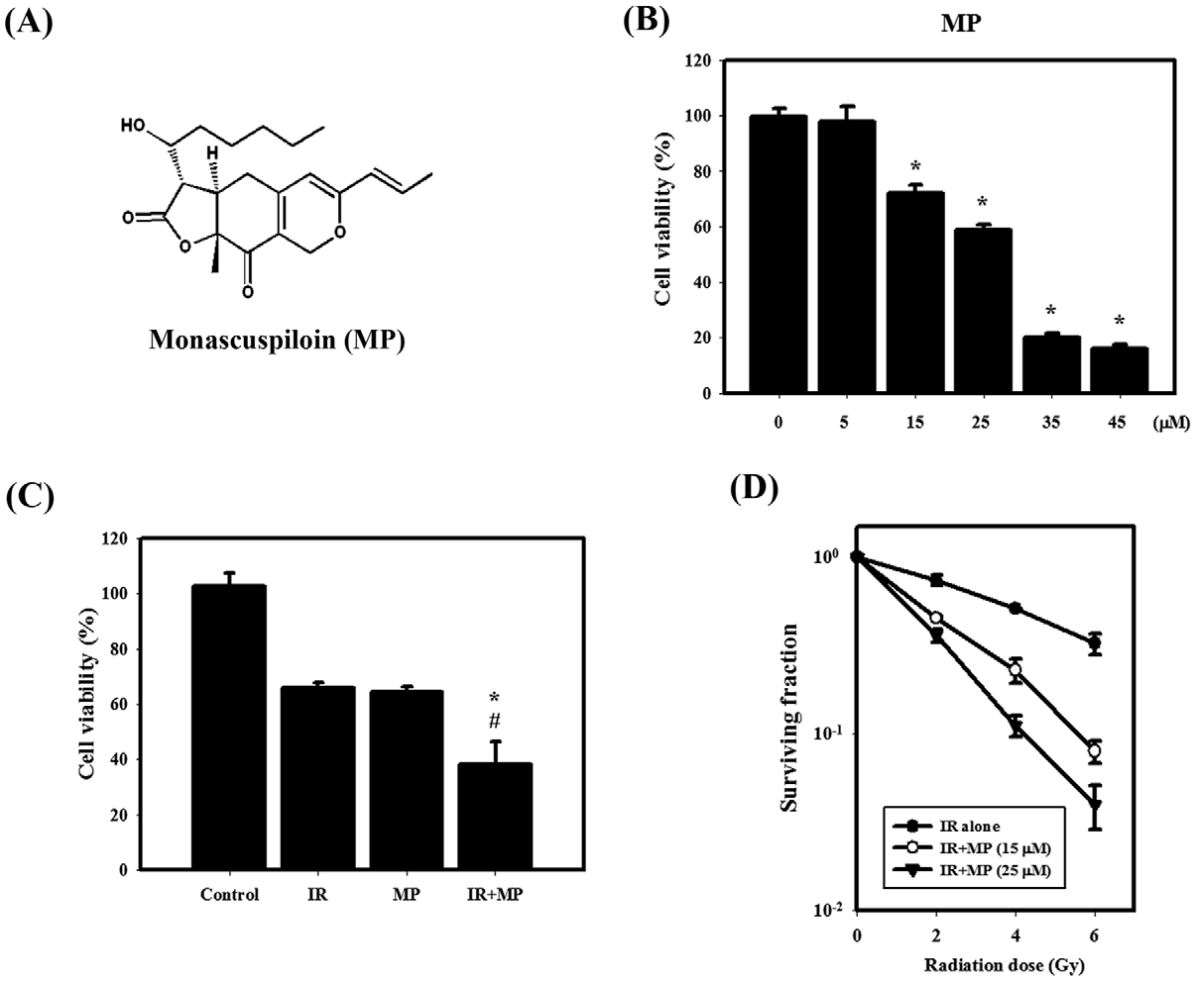
IR dose–response survival curves and cytotoxic effects resulting from MP and/or IR in PC-3 cells. (A) Chemical structure of MP. (B) Concentration-dependent effects of MP on the viability of PC-3 cells. Cells were treated with 5, 15, 25, 35 or 45 μM MP for 48 hrs. *, *p*<0.05, MP versus control. (C) Cytotoxic effects in cells treated with IR (4 Gy) and/or MP (25 μM). #, *p*<0.05, IR versus combined treatment. *, *p*<0.05, MP versus combined treatment. (D) The radiation dose-response survival curves of PC-3 cells with or without MP. Data are presented as the mean ± standard deviation of three independent experiments.

Monascuspiloin (MP), a yellow pigment first isolated by our group from *Monascus pilosus M93*-fermented rice, is a structurally similar to the well-known *Monascus* pigment monascin. However, the mechanisms underlying the effects of MP in combination with IR on prostate cancer are largely unknown. In the present study, PC-3 cells (androgen-independent human prostate cancer cells) were used to investigate the anti-cancer effects of IR combined with MP *in vitro* and *in vivo*. The types of cell death induced by IR when combined with MP were examined. We also investigated the possible mechanisms underlying cell death induced by IR and/or MP in the PC-3 cells.

**Figure 2:**
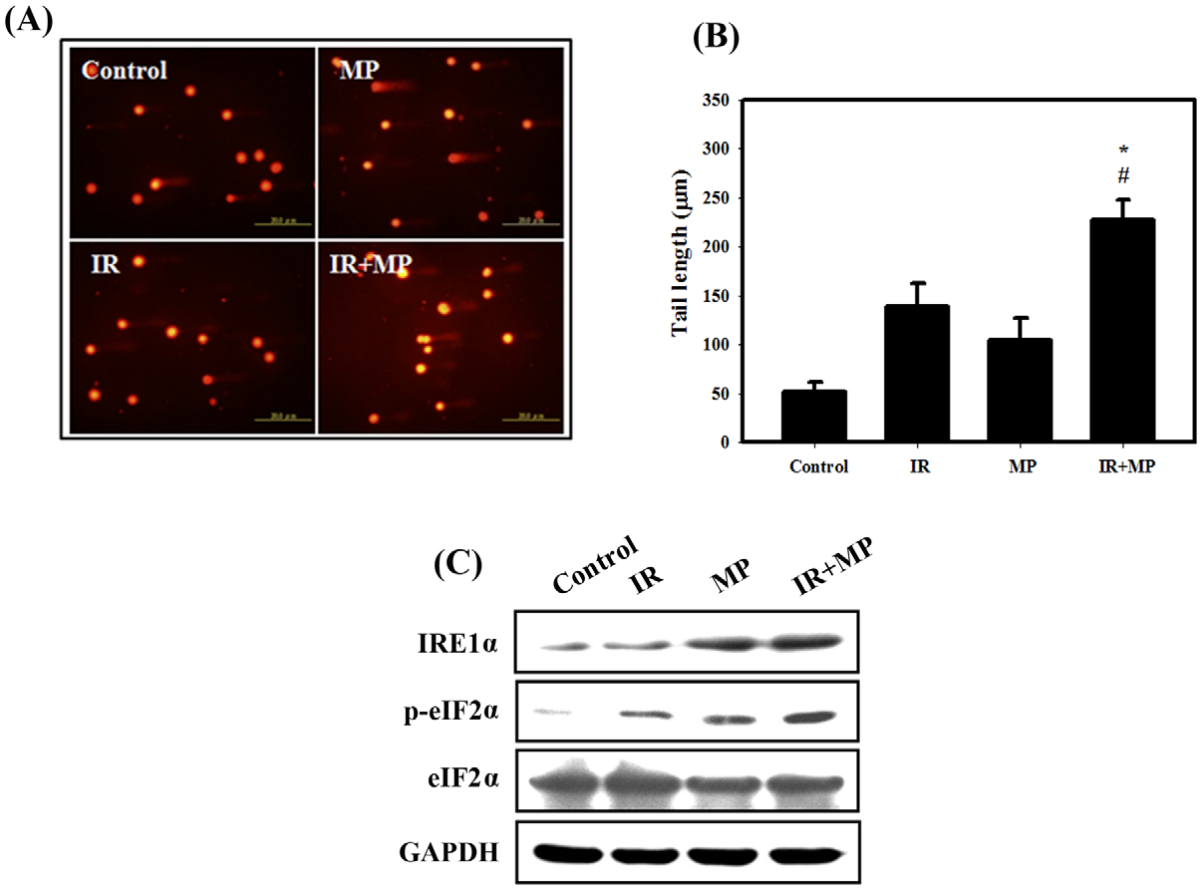
Comet assay and ER stress induced by IR and/or MP in PC-3 cells. (A) EtBr staining of cells treated with IR (4 **Gy) and MP (25** **μM).** The tails indicate DNA damage. (B) The effect of MP or IR alone or in combination for 48 hrs on average tail DNA. #, *p*<0.05, IR versus combined treatment. *, *p*<0.05, MP versus combined treatment.

## Materials and Methods

### Cell culture

The human prostate cancer cell line PC-3 (ATCC CRL-1435) was obtained from the American Type Culture Collection (ATCC). The cells were cultured in RPMI 1640 medium (Gibco BRL, Grand Island, NY) supplemented with antibiotics containing 100 U/ml penicillin, 100 μg/ml streptomycin (Gibco BRL, Grand Island, NY) and 10% fetal bovine serum (HyClone, South Logan, UT, USA). The cells were incubated in a humidified atmosphere containing 5% CO_2_ at 37°C. Exponentially growing cells were detached using 0.05% trypsin-EDTA (Gibco BRL, Grand Island, NY) in RPMI 1640 medium.

**Figure 3:**
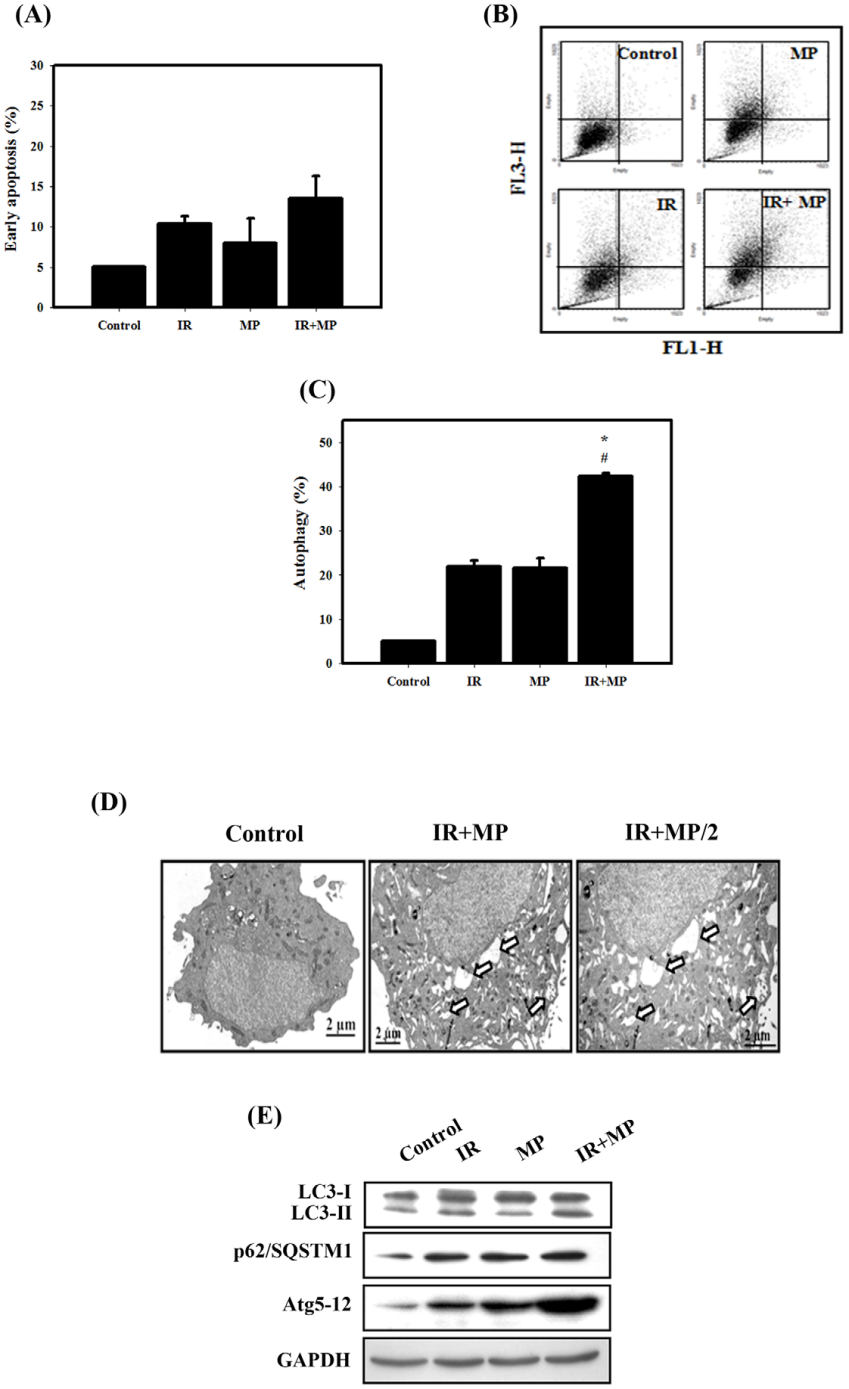
Measurement of apoptosis and autophagy in PC-3 cells that received various treatments. (A) Early apoptosis, detected using an Annexin V apoptosis detection kit, was measured using flow cytometry. Cells were treated with IR (4 Gy) and MP (25 μM) for 48 hrs. (B) Development of AVOs in PC-3 cells. Detection of green and red fluorescence in AO-stained cells using flow cytometry. (C) Quantification of AVOs with AO-stained cells treated with IR (4 Gy) or MP (25 μM) alone or in combination using flow cytometry. #, *p*<0.05, IR versus combined treatment. *, *p*<0.05, MP versus combined treatment. Data are presented as the mean ± standard deviation of three independent experiments. (D) EM microphotographs of PC-3 cells treated with IR (4 Gy) and MP (25 μM) for 48 hrs. The *white arrows* point to autophagic vacuoles and autolysosomes. (E) Western blotting for LC3-I, LC3-II, p62/SQSTM1 and Atg5–12 in PC-3 cells. Cells were treated with IR (4 Gy) and MP (25 μM) for 48 hrs.

### Microorganism and preparation of monascuspiloin (MP)

*Monascus pilosus M93* was obtained from BCRC, FIRDI (Hsinchu, Taiwan) and maintained on potato dextrose agar (PDA; Difco). The strain was plated onto PDA plates and cultivated at 25°C for 7 days. The spores were then washed out from the PDA plate using sterile water and the concentration of the resulting spore suspension was adjusted to 1×10^6^/ml. Following the spores enrichment step, 1 ml spore suspension were inoculated into 250 ml shake flasks containing 50 ml RGY medium (3% rice starch, 7% glycerol, 1.2% polypeptone, 3% soybean powder, 0.1% MgSO_4_ and 0.2% NaNO_3_) and cultivated with shaking (150 rpm) at 25°C for 3 days to obtain the mycelium broth of M93. For the production of RYR, fifty 450-ml glass bottles, each containing 75 g rice and 75 ml D.I. water, were sterilized for 20 min at 121°C. M93 mycelium broth (7.5 ml) and RGY medium (7.5 ml) were added to each bottle. The bottltes were then incubated at 25°C for 21 days (7.5 ml RGY medium was added to each bottle on the 10^th^ day of incubation), and the contents were then lyophilized to remove water. The RYR (1 kg) was extracted using 95% ethanol (3 L) at 25°C for 24 hrs, and the ethanol was removed by vacuum-drying to obtain the crude RYR extract. The amount of MP in the crude extracts was measured using HPLC with a purospher® star RP-18 column (Merck). The mobile phase comprised 60% acetonitrile containing 0.1% phosphate at flow rate of 1.0 ml/min. The RYR (1 kg) was then extracted with 70% ethanol (3 L) at 25°C. After filtration, the ethanol extract was concentrated and ethyl acetate (EA) was added to obtain an EA-soluble fraction. The EA-soluble fraction was injection into a silica gel column (70–230, 230–400 mesh, Merck) and eluted using n-hexane/ethyl acetate (2∶1). To obtain MP, the eluted fraction was concentrated under reduced pressure and purified using preparative thin-layer chromatography (silica gel 60 F-254, Merck) with *n*-hexane/EtOAc(2∶1).

**Figure 4:**
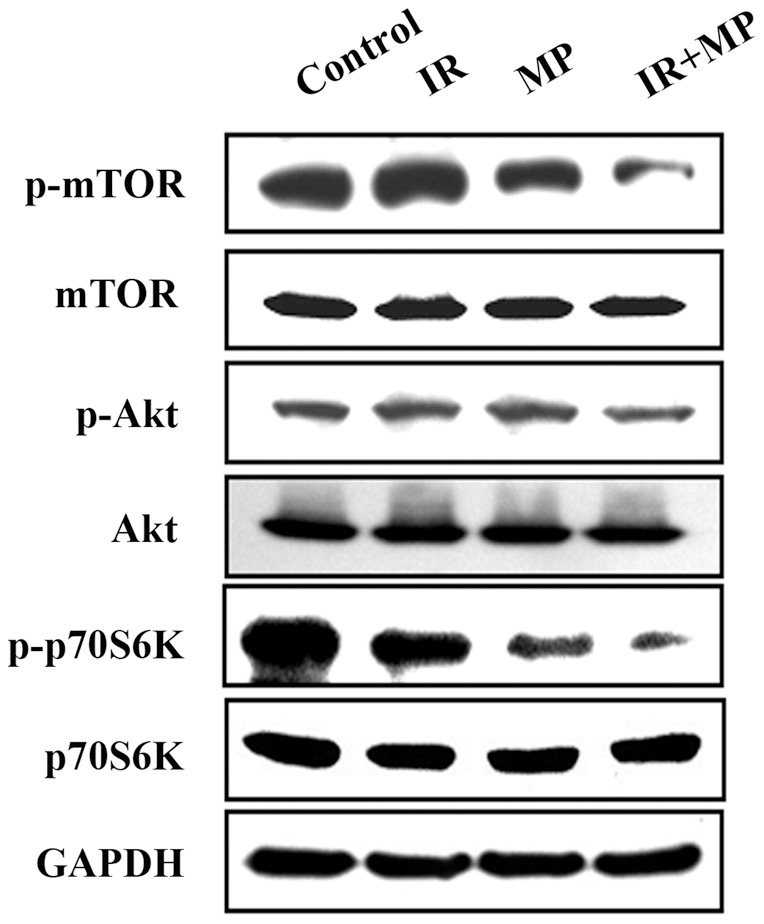
Akt/mTOR signaling pathway protein expression in PC-3 cells treated with IR and MP alone or in combination. Cells were treated with IR (4 **Gy) and/or MP (25** **μM) for 48** **hrs.**

Optical rotations were measured using a Jasco P-1020 digital polarimeter. UV spectra were obtained in MeOH using a Jasco UV-240 spectrophotometer, and IR spectra (KBr or neat) were obtained using a Perkin-Elmer System 2000 FT-IR spectrometer. The 1D (^1^H, ^13^C, DEPT) and 2D (COSY, NOESY, HSQC, HMBC) NMR spectra using CDCl_3_ and CD_3_OD as solvents, were recorded using a Varian Unity Plus 400 (400 MHz for ^1^H NMR, 100 MHz for ^13^C NMR) and Varian INOVA-500 (500 MHz for ^1^H NMR, 125 MHz for ^13^C NMR) spectrometer. Chemical shifts were referenced to the internal solvent signals in CDCl_3_ (^1^H, δ 7.26; ^13^C, δ 77.0), and TMS was used as the internal standard. Low-resolution ESI-MS spectra were obtained using an API 3000 (Applied Biosystems), and high-resolution ESI-MS spectra were obstained using a Bruker Daltonics APEX II 30e spectrometer.

**Figure 5:**
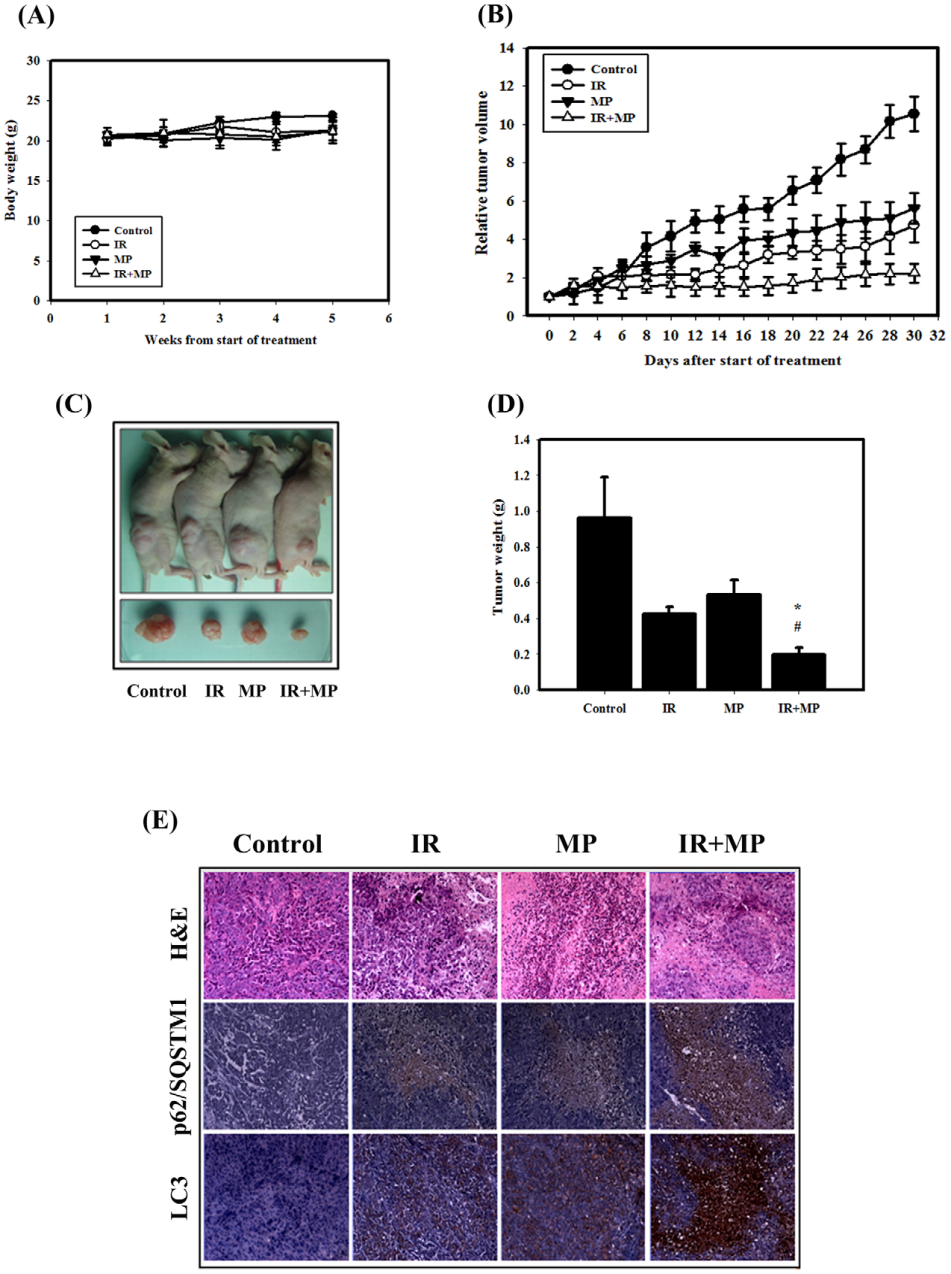
Tumor growth and body weight of tumor-bearing mice treated with IR (6 **Gy) or MP (1** **mg/kg×6) alone or in combination.** (A) Body weight in nude mice, measured once per week. (B) Tumor volume in nude mice, measured every two days. Data are presented as the relative tumor volume (mean ± standard error) normalized to the initial tumor volume measured on day 0. (C) Direct observations of mice with tumors. Following experiments, the mice were sacrificed and the tumors were removed. (D) Measurement of tumor weight in the nude mice after sacrifice. (E) Immunohistochemical (IHC) and hematoxylin and eosin (H&E) staining of PC-3 mouse xenograft tissues. IHC was used to determine the expression levels of LC3 and p62/SQSTM1 (×100 objective magnification).

**Table 1:** Comparison of tumor growth inhibition, tumor volume quadrupling time, and tumor growth delay time of PC-3 tumors in nude mice.

	Number of mice	Inhibition*(%)	Tumor volume quadrupling time (days)	Tumor growth delay time (days)	p value (t test)^#^ Control IR MP		
**Control**	**5**	**-**	**10.3±2.4**	**-**			
**IR (6Gy)**	**5**	**52.4±11.3**	**28.1±6.2**	**17.8±6.2**	**<0.05**		
**MP (1mg/kg × 6)**	**5**	**42.5.4±12.6**	**20.7±4.2**	**10.4±4.2**	**<0.05**	**0.74**	
**IR+MP**	**5**	**71.4± 8.1**	**62.6±7.1**	**52.3±7.1**	**<0.05**	**0.07**	**<0.05**

* **Tumor growth inhibition rate was calculated based on the tumor volume on ay 10.**

**#** ***p*** **values for comparison of tumor growth delay time.**

MP, isolated as a yellowish oil, was assigned the molecular formula C_21_H_28_O_5_Na by ESI-MS ([M+Na]^+^, *m/z* 383) and HR-ESI-MS ([M+Na]^+^, *m/z* 383.1832). MP was similar to that of a known compound, monascin, the structure of MP was determined to be (3*S*,3a*R*,9a*R*)-3a,4-dihydro-3-((*S*)-1-hydroxyhexyl)-9a-methyl-6-((E)-prop-1-enyl)-3*H*-furo [3, 2-*g*]isochromene-2,9(8*H*,9a*H*)-dione (Fig. 1A).

### Irradiation treatment and cell viability assay

IR was performed with 6 MV X-rays using a linear accelerator (Digital M Mevatron Accelerator, Siemens Medical Systems, CA, USA) at a dose rate of 5 Gy/min. An additional 2 cm of a tissue-equivalent bolus was placed on the top of the plastic tissue-culture flasks to ensure electronic equilibrium, and 10 cm of tissue-equivalent material was placed under the flasks to obtain full back-scatter. The treated cells were centrifuged and resuspended in 0.1 ml PBS. Each cell suspension (0.02 ml) was mixed with 0.02 ml trypan blue solution (0.2% in PBS). After 1 or 2 min, each solution was placed on a hemocytometer, and the blue stained cells were counted as non-intact.

### Clonogenic assay

The cells were irradiated using 2, 4 or 6 Gy. MP was added to the cells at concentrations of 15 or 25 μM. The cells were trypsinized and counted. Known numbers of cells were subsequently replated in 6-cm culture dishes and returned to the incubator to allow for colony development. After 7 days, colonies (containing ≥50 cells) were stained with 0.5% crystal violet solution for 30 min. The plating efficiency (PE) is the ratio of the number of colonies to the number of cells seeded in the nonirradiated group. Calculation of survival fractions (SFs) was performed using the equation: SF  =  colonies counted/(cells seeded ×PE), using the individual PE.

### Determination of early apoptosis

Apoptosis was assessed by quantifying the translocation of phosphatidyl serine to the cell surface, detected with an Annexin V apoptosis detection kit (Calbiochem, San Diego, CA, USA), according to our previous report [23].

### Comet assay

To detect DNA damage in individual cells, we adopted a comet assay. Briefly, cells (2×10^4^) were collected after the treatments and resuspended in 0.2 ml PBS containing 0.5% low-melting-point agarose. Eighty-five microliters of the mixture was applied to slides, which were then submerged in cold lysis solution (2.5 M NaCl, 0.1 M EDTA, 10 mM Tris, 1% Triton X-100 (pH 10)). Electrophoresis was performed at 300 mA and 25 V for 20 min. After electrophoresis, the slides were neutralized with 0.4 M cold Tris-HCl buffer (pH 7.5) and then stained using ethidium bromide. Comets were visualized using a fluorescence microscope (Olympus, Japan). DNA damage was assessed in 100 cells where tail moment (tail length multiplied by the fraction of DNA in the tail) was quantified using Image-Pro Plus (Media Cybernetics Inc.).

### Supravital cell staining with acridine orange for detection of autophagy

Cell staining with acridine orange (Sigma Chemical Co.) was performed according to published procedures [23].

### Electron microscopy

Cells were fixed using a solution containing 2.5% glutaraldehyde and 2% paraformaldehyde in 0.1 M cacodylate buffer, pH 7.3, for 1 hr. After fixation, the samples were postfixed in 1% OsO_4_ in the same buffer for 30 min. Ultra-thin sections were subsequently observed under a transmission electron microscope (JEOL JEM-1200EX, Japan) at 100 kV.

### Western blot analysis

Total cellular protein lysates were prepared by harvesting cells in protein extraction buffer for 1 hr at 4°C, as described previously [24]. The densities of the bands were quantified using a computer densitometer (AlphaImager^TM^ 2200 System Alpha Innotech Corporation, San Leandro, CA, USA). GAPDH was used as the protein loading control. Antibodies for detecting Akt, phospho-Akt, phospho-mTOR, phospho-AMPK, AMPK and phospho-p70S6K were obtained from Cell Signaling Technology (Ipswich, MA, USA); anti-GAPDH was obtained from Abcam (Cambridge, MA, USA); anti-LC3 and anti-Atg5-12 were obtained from Abgent (San Diego, CA, USA); anti-p62/SQSTM1 antibody was obtained from MBL (Nagoya, Japan), and anti-mTOR and anti-p70S6K were obtained from Epitomics (Burlingame, CA, USA).

### In vivo tumor growth assays using the PC-3 tumor model in nude mice

All experiments on mice were performed according to the guidelines of our institute (the Guide for Care and Use of Laboratory Animals, National Cheng Kung University). The animal use protocol listed below has been reviewed and approved by the Institutional Animal Care and Use Committee (IACUC) (Approval No: 99138). Six- to eight week-old male nude mice (BALB/cAnN.Cg-Foxn1^nu^/CrlNarl) were acquired from the National Laboratory Animal Center (Taiwan). The animals were housed five per cage at 24±2°C and 50%±10% relative humidity and subjected to a 12-h light/12-h dark cycle. The animals were acclimatized for 1 week prior to the start of experiments and fed with a Purina chow diet and water ad libitum. Tumors were induced by subcutaneous (s.c.) injection of PC-3 cells (2×10^6^ cells in 0.1 ml of PBS) at one site of the right flank. Tumors (visualized as small nodules at the sites of injection) appeared ∼7 days after injection, and the animals were randomly distributed into each group. Mice were treated with: (1) vehicle (corn oil), (2) 1 mg/kg MP three times per week for two weeks, (3) a single dose of 6 Gy IR, or (4) a combination treatment comprising 1 mg/kg MP three times per week for two weeks initiated immediately after a single dose of 6 Gy IR. Mouse body weights were measured once per week and were used as an indicator of systemic toxicity of the treatment. Tumor growth was measured every two days, and the tumor volume was calculated according to the formula shown below [25]:

Tumor volume (mm^3^)  =  larger diameter (mm) × small diameter (mm) ^2^/2

Data are expressed as the relative tumor volume compared to the pretreatment volume measured on day 0. The tumor volume quadrupling time (TVQT, in days) was determined by a best-fit regression analysis. The tumor growth delay (TGD) time is defined as the difference between the TVQT of the treated tumors compared with that of untreated control tumors. The TVQT and TGD time were calculated for each individual mouse, and then the means were generated for each group. Tumor growth inhibition rate was calculated as follows: Inhibition (%)  =  (mean tumor volume of untreated control mice – tumor volume of treated mice)/mean tumor volume of untreated control mice ×100.

### Immunohistochemical (IHC) staining analysis

Paraffin-embedded tissue sections (4 μm) were dried, deparaffinized, and rehydrated. Following microwave pretreatment in citrate buffer (pH 6.0; for antigen retrieval), the slides were immersed in 3% hydrogen peroxide for 20 min to block the activity of endogenous peroxidase. After intensive washing with PBS, the slides were incubated overnight at 4°C with the anti-LC3 and anti-p62/SQSTM1 (MBL, Japan) antibodies. The sections were then incubated with a secondary antibody for 1 hr at room temperature, and the slides were developed using the STARR TREK Universal HRP detection kit (Biocare Medical, Concord, CA). Finally, the slides were counterstained using hematoxylin. Each slide was scanned at low power (× 200).

### Statistical analysis

Data are expressed as the mean ± SD. Statistical significance was determined using Student's t-test for comparison between the means or one-way analysis of variance with post-hoc Dunnett's test [26]. Differences were considered to be significant when p<0.05.

## Results

### Cytotoxic effects and survival curves of MP and IR treated alone or in combination on PC-3 cells

The viability of the cells was observed at different concentrations of MP (0 to 45 μM) for 48 hrs (Fig. 1B). MP alone reduced the viability of cells in a concentration-dependent manner. After treatment with 25 μM MP for 48 hrs, the viability of PC-3 cells was decreased to 60%. Figure 1C shows the viability of MP and IR treated alone or in combination on PC-3 cells. Significantly enhanced toxicity was found for the combined treatment compared with MP and IR treatment alone in PC-3 cells. Furthermore, figure 1D shows the radiation dose–response survival curves for PC-3 cells with or without MP treatment. The survival curves dramatically shifted downward. MP (15 or 25 μM) increased IR-induced clonogenic cell death in PC-3 cells. MP significantly reduced the survival fraction in a dose-dependent manner compared to IR alone.

### DNA damage determined by the comet assay and expression of ER stress-related proteins in PC-3 cells treated with MP and IR alone or in combination

IR plays a key role in cancer therapy due to its ability to directly induce DNA damage [27]. The comet assay is a sensitive tool for the estimation of DNA damage and repair at the cellular level, requiring only a small number of cells [28]. In the comet assay for the PC-3 cells, a limited or no tail was found in the untreated controls, while the tail grew after irradiation and combined treatment (Fig. 2A). The tail length grew significantly after IR (4 Gy) compared to controls (Fig. 2B). Significantly enhanced DNA damage was found for the combined treatment compared with IR alone. These results suggested that DNA damage was involved in the anti-proliferative effects of combined treatment in PC-3 cells. In addition, recent evidence shows that one of the mechanisms whereby IR activates ER stress and the UPR is by the induction of DNA damage [29]. Therefore, to detect the expression of ER stress-related proteins, we performed western blotting with lysates from PC-3 cells receiving each of the different treatments (Fig. 2C). Our results showed that IRE1α and phosphorylation of eIF2α increased with combined treatment compared with MP or IR treatment alone, thus confirming that the combined treatment induced ER stress in PC-3 cells.

### Measurement of apoptosis and autophagy in PC-3 cells treated with MP and IR alone or in combination

As shown in Figure 3A, early apoptosis in PC-3 cells was measured by flow cytometry with the Annexin V apoptosis detection kit. Quantitative results showed that the occurrence of early apoptosis in PC-3 cells treated with MP and/or IR was low. Thus, we further analyzed that type II programmed cell death (autophagy). It is characterized by the formation of numerous acidic vesicles, which are called acidic vesicular organelles (AVOs) [30]. Acridine orange (AO) staining was quantified using flow cytometry (Figs. 3B, 3C). A significant increase in AO-positive cells was found for cells receiving the combined treatment with compared to those treated with IR or MP alone. Furthermore, the ultrastructures of the PC-3 cells for each treatment group were observed by EM photomicrography (Fig. 3D). The combined treatment also resulted in a large number of autophagic vacuoles and autolysosomes in the cytoplasm. In addition, there was no chromatin condensation or nuclear pyknosis, which are characteristic of apoptosis, occurred in any of treated cells. To detect the expression of autophagy-related proteins, we performed western blotting with lysates from PC-3 cells receiving each of the different treatments (Fig. 3E). The expression levels of LC3-II, p62 and Atg5-12 proteins increased with combined treatment, suggesting that the combined treatment induced autophagy in PC-3 cells.

### The Akt/mTOR signaling pathway is involved in the combined treatment-induced autophagy in PC-3 cells

Previous studies have demonstrated that the Akt/mTOR pathway is involved in regulating autophagy [31]. Therefore, to investigate whether the Akt/mTOR signaling pathway was involved in the combined treatment-induced autophagy, we performed western blotting to detect the protein phosphorylation state (Fig. 4). Phosphorylated proteins that are involved in the Akt/mTOR signaling pathways were also examined. Phosphorylation of Akt, mTOR and p70S6K decreased in cells treated with the combined treatment compared with MP or IR treatment alone.

### Tumor growth in nude mice was suppressed by IR and MP

We next evaluated the anti-tumor growth effect of IR and MP *in vivo*. Tumors were induced by s.c. injection of PC-3 cells into nude mice. We measured the body weight of the mice on weekly basis and the tumor volume every second day. The results of this study demonstrated that none of the treatment regimens produced any loss of body weight, which may be a sign of toxicity (Fig. 5A). The combined treatment suppressed tumor volume and tumor weight in nude mice compared with MP or IR treatments alone (Figs. 5B, 5C, 5D). As shown in Table 1, the tumor volume quadrupling time (TVQT) of the control group was measured on Day 10 (10.3±2.4). The tumor growth delay (TGD) time for the 10 day treatment of MP alone was 10.4±4.2 days, which was significantly different than for the control group (*p<*0.05). IR alone produced a TGD time of 17.8±6.2 days (*p<*0.05 compared to the control group). The combined treatment resulted in a TGD time of 52.3±7.1 days (*p<*0.05, compared with control group or MP alone). The combination therapy of IR and MP resulted in a tumor growth inhibition of 71% on Day 10. Thus, the combined treatment possesses anti-tumor growth effect *in vivo*.

Next, histological examination were analyzed by hematoxylin and eosin (H&E) staining (Fig. 5E). The tumor of combined treatment was composed of cells with the lower nucleus-to-cytoplasm ratios than MP or IR treatment alone. Furthermore, the LC3 and p62 expression patterns in PC-3 tumors were examined using IHC staining. LC3 and p62 were increased in tumors from mice treated with the combined treatment compared with MP or IR treatment alone.

## Discussion

Recently, it was reported that RYR, a traditional Chinese food herb and modern dietary supplement, demonstrated *in vitro* effects, including inhibition of proliferation and stimulation of apoptosis in human colon cancer cells [6]. In addition, RYR significantly reduced tumor volumes of prostate xenograft tumors [32]. The combination of IR with other agents that achieve radiosensitizing potential has become important interventions for the patients with prostate cancer [33]. Previous studies have also demonstrated that natural products as the novel radiosensitizers for the treatment of hormone-refractory prostate cancer that is resistant to radiation therapy [11], [34]. In the present study, we demonstrated for the first time that MP sensitizes human prostate cancer PC-3 cells to IR both *in vitro* and *in vivo* in the nude mouse xenograft model (Figs. 1, 5). For most of the history of cancer therapy, apoptosis was thought to be the only mechanism of drug-induced cell death. More recently, it has been reported that a type II programmed cell death, autophagy, may participate in cancer therapy-induced cancer cell death. Recent findings, including our laboratory, have suggested that activated autophagy is a tumor suppressor [23], [24], [35]. Here, the PC-3 cells treated with combined treatment induced autophagy potentially occurred when the apoptotic pathway of PC-3 cells was blocked (Fig. 3). In the *in vivo* study of PC-3 xenograft tumors, LC3 and p62 were increased following the combined treatment (Fig. 5E).

Radiation plays a key role in therapy due to its ability to directly induce DNA damage, particularly DNA double-strand breaks, thus leading to cell death [27]. In our current study, significantly enhanced DNA damage was found in the combined treatment group compared with IR or MP alone (Figs. 2A, 2B). However, the detail of the mechanism is not yet fully understood. It is possible that MP interferes with the processing and repair of IR-induced DNA double-strand breaks. Alternatively, MP might interfere with a post-repair step involving the actual reversal of γH2AX, such as dephosphorylation or degradation and/or a histone exchange of γH2AX [36]. Recent evidence shows that one of the mechanisms whereby IR activates ER stress is the induction of DNA damage [29]. Thus, double stranded DNA breaks may serve as one link between IR and ER stress activation. Our results showed that IRE1α and phosphorylation of eIF2α increased with combined treatment, indicating that combined treatment induced ER stress in PC-3 cells (Fig. 2C). ER-stress can activate signaling pathways involved in apoptosis and autophagy [22]. In mammalian cells, ER stress has been shown to facilitate the formation of autophagosomes, and induction of autophagy enables the removal of toxic misfolded proteins [37]. Ullman et al. reported that autophagy is invoked as a means of killing cells when ER stress is implacable [38]. A better understanding of the signaling pathways controlling autophagy and ER stress will hopefully open new possibilities for the treatment of the numerous diseases associated with ER stress.

The Akt/mTOR pathway plays a crucial role in the regulation of both apoptosis and autophagy. Disruption of the PI3K/Akt pathway, culminating in Akt inhibition, has been found to be associated with autophagy induced by a variety of antineoplastic agents in cancer cells [39]. Viola et al. indicated that MG-2477, a new tubulin inhibitor, induces autophagy via inhibition of the Akt/mTOR pathway in A549 cells [40]. Triptolide induces autophagy in pancreatic cancer cells and also inhibits the Akt/mTOR/p70S6K pathway [41]. Here, we demonstrate that the expression of p-Akt, p-mTOR and p-p70S6K proteins decreased in cells treated with the combined treatment compared to treatment with MP or IR alone (Fig. 4). These results suggest that Akt inhibition may be one of the common mechanisms of autophagy induction by anticancer agents.

To our knowledge, this is the first report on the combined application of MP with IR for human prostate cancer therapy. Our results indicate that IR combined with MP increases the therapeutic efficacy compared to individual treatments in PC-3 human prostate cancer cells. Specifically, the combined treatment induced autophagy, ER stress and enhanced DNA damage in PC-3 cells. The combined treatment-induced autophagy occurred primarily via inhibition of the Akt/mTOR signaling pathways. In a nude mouse tumor xenograft model, the combination treatment possesses an anti-tumor growth effect. IR combined with MP could provide a novel therapy for the treatment of androgen-independent prostate cancer.
